# Multilocus inherited neoplasia allele syndrome: report of uncommon combinations between *CHEK2/ATM* and *BRCA1/CDKN2A* genes

**DOI:** 10.3332/ecancer.2024.1701

**Published:** 2024-05-10

**Authors:** Ricardo Ubilla, Michelle Zeppelin, Fernanda Martin

**Affiliations:** 1 Departamento de Genética, Hospital Luis Calvo Mackenna, Santiago 7500539, Chile; 2 Unidad Genética, Hospital Clínico Universidad de Chile, Santiago 8380456, Chile; 3 Unidad Asesoramiento Genético Oncológico, Fundación Arturo López Pérez, Santiago 7500691, Chile

**Keywords:** MINAS, CHEK2, ATM, BRCA1, CDKN2A

## Abstract

**Background:**

Multilocus inherited neoplasia allelic syndrome (MINAS) is a recently coined term that describes the coexistence of two or more pathogenic variants (PVs) in cancer susceptibility genes (CSGs) in a single individual.

**Case presentation:**

This article presents two cases of MINAS due to rare CSG combinations. The first was a 37-year-old woman carrying PVs in the mutated ataxia telangiectasia (*ATM*) and *CHEK2* genes, with HER-2 positive unilateral breast cancer at 29. The second was a 53-year-old woman carrying PVs in the *BRCA1* and *CDKN2A* genes, who presented with triple-negative breast cancer at 51. We describe their family history and treatment, where the lack of evidence for personalised management becomes evident.

**Conclusion:**

Predicting the phenotypic effect of harbouring two variants in CSG is challenging. It is essential to encourage the notification of other cases and carry out functional studies to establish specific risks for affected individuals to develop personalised follow-up guidelines to reduce the associated morbimortality.

## Background

The development and progression of cancer are usually the product of somatically acquired genetic and epigenetic events. However, between 5% and 10% of cases are explained by germline genetic variants in genes related to the control of cell proliferation or cell death cancer susceptibility genes or (CSG) that act as drivers in the process, resulting in a hereditary predisposition to the development of cancer [1]. The frequency of hereditary cancers varies among different types of cancer, selection criteria for testing and ethnicities, with the highest prevalence of pathogenic variants (PVs) found in ovarian cancer around 15%–20% [2]. Carrying germline variants in these genes in the general population is considered a rare event, and the probability that an individual has a PVs in more than one CSG is even lower. Its occurrence is named ‘multilocus inherited neoplasia allele syndrome’ (MINAS), a term coined in 2016 by Whitworth *et al* [3]. Reports of MINAS have increased in recent years due to the wider availability of next generation sequencing (NGS) technology and broader genetic studies for cancer. McGuigan *et al* [4] published one of the most comprehensive reviews on the syndrome reporting 385 cases published in the literature between 1996 and 2020. From the aforementioned, 78.5% correspond to individuals who carry *BRCA1/2* variants and only two cases (1.03%) presented variants in mutated ataxia telangiectasia (*ATM*) and *CHEK2*. To the best of our knowledge, no cases of patients with simultaneous PVs in *BRCA1* and *CDKN2A* are reported in the literature [4, 5].

Except for MINAS caused by variants in *BRCA*1/2, information on the possible implications of combinations of PVs in other genes for the risk of developing tumours is limited.

We herein present two cases of MINAS due to uncommon combinations of CSGs.

### Case 1: ATM + CHEK2

We present the case of a 37-year-old daughter of non-consanguineous parents with a history of pure HER2-positive right breast cancer, diagnosed at 29. She was treated with a modified radical mastectomy of the right breast, adjuvant chemotherapy with trastuzumab, radiotherapy and breast reconstruction.

In addition, she had a history of hypothyroidism under treatment, iron deficiency anaemia, abnormal uterine bleeding on follow-up and three first-trimester abortions without an etiological study.

**Supplementary Figure 1.**
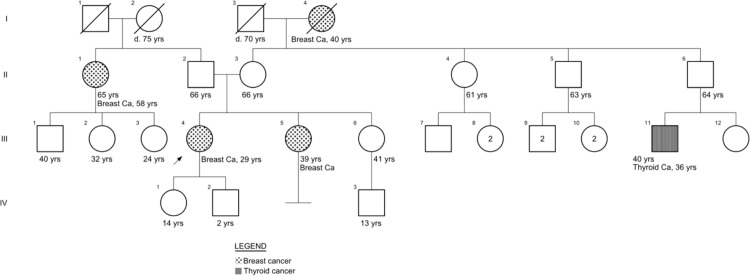
Family pedigree case 1.

She had a familial history of breast cancer from her maternal grandmother and a paternal aunt, diagnosed at 40 and 65 years of age, respectively, and a paternal cousin with thyroid cancer (of unknown histology) at 36. Her sister presented with a suspicious breast lump at age 39, with a biopsy reporting a tubular adenoma (Supplementary Figure 1).

At 37, she consulted in an oncological genetic counseling unit, where an NGS panel of 36 genes associated with breast and ovarian was solicited. A heterozygous PV in *ATM* (NM_000051.3):c.2466+1G>A (splicing donor) and a likely pathogenic heterozygous variant in *CHEK2* (NM_007194.3):c.445-2A>G (splicing acceptor) were identified, compatible with a MINAS diagnosis. GRCh37 was used as the reference genome. Cascade family testing of both variants was not performed due to economic reasons.

Regarding other tests, she had a colonoscopy with only external hemorrhoids at 38, a normal Pap smear at 38, and a transvaginal ultrasound requested for abnormal uterine bleeding at 38 years of age, which reported heterogeneous myometrium, 24-mm thickened endometrium, normal ovaries and signs of adenomyosis. Her last contralateral mammography and breast echography had normal results.

After post-test counseling of the genetic test result, follow-up recommendations for each gene were delivered according to the National Comprehensive Cancer Network (NCCN) guidelines [6]. The patient decided to opt for a risk-reducing mastectomy of the left breast and consideration of bilateral salpingo-oophorectomy in conjunction with eventual hysterectomy indicated according to endometrial biopsy. A karyotype was solicited due to her history of recurrent abortion; however, it was not done by the time of writing this paper.

### Case 2: BRCA1 + CDKN2A

The second case was a 53-year-old female, daughter of non-consanguineous parents, with a history of triple-negative breast cancer, diagnosed at 51 years of age. In addition, she had a history of non-insulin-requiring diabetes mellitus under treatment.

**Supplementary Figure 2.**
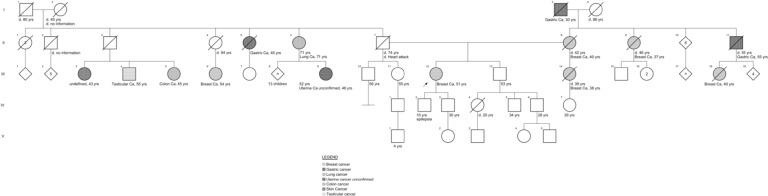
Family pedigree case 2.

She had a family history of breast cancer from her mother, half-sister (maternal), maternal aunt and cousin and paternal cousin diagnosed at 40, 38, 37, 40 and 54 years, respectively (Supplementary Figure 2). Also, her maternal grandfather and paternal uncle had gastric cancer diagnosed at 30 and 40 years of age, respectively; her paternal aunt was diagnosed with lung cancer at age 71; a paternal cousin with colon cancer diagnosed at age 45; a paternal cousin with testicular cancer diagnosed at age 55 and a paternal cousin diagnosed with skin cancer at age 43.

At 52, she consulted in an oncological genetic counseling unit, where an NGS multi-cancer panel of 84 genes was solicited. The result was the identification of a heterozygous PV in *BRCA1*(NM_007294.4):c.3759dup (p.Lys1254*), associated with hereditary breast-ovarian cancer syndrome and a novel heterozygous PV in *CDKN2A* (NM_000077.4, p16INK4A):c.-34G>T associated with familial atypical multiple mole melanoma syndrome (FAMMMS). GRCh38 was used as the reference genome. Cascade family testing of both variants was not performed for economic reasons.

Follow-up recommendations related to each gene were delivered according to the NCCN guidelines [6] and the International Cancer of the Pancreas Screening consortium [7]. She underwent neoadjuvant chemotherapy, had a bilateral mastectomy and salpingo-oophorectomy, and was referred for dermatology and gastroenterology follow-up. As for her skin phenotype, she had a normal presence of nevi with less than 50 nevi.

## Discussion

We presented two cases of patients diagnosed with MINAS due to PVs in the *ATM*/*CHEK2* and *BRCA1/CDKN2A genes*. To the best of our knowledge, Case 1 corresponds to the fifth published MINAS case with this gene combination and Case 2 is the first case reported with *BRCA1/CDKN2A* variants [4, 5].

In previous cases with *ATM/CHEK2* combination (all female), two presented only breast cancer (diagnosed at 39 and 60 years), one presented breast and bladder cancer (diagnosed at 53 and 58 years, respectively), and one patient presented colorectal cancer at 44 [8–10].

From the phenotype perspective, it has been postulated that the coexistence of two PVs in different CSG could have additive consequences, where the risk of developing neoplasms reflects the risks provided by each gene independently. Synergistically, where the effects are more severe than those observed when only carrying one variant (in age of onset or type of tumour) [4, 11]. Some factors that could favour synergism between CSGs include the involvement of two oncogenes, location in nearby chromosomal regions, and participation in the same tumourigenic pathways [3, 4]. Notably, atypical tumour phenotypes appear in approximately 15% of non-*BRCA1/2* MINAS [4].

The ATM-CHEK2-P53 signaling pathway is one of the main pathways to preventing genomic instability and cancer development by participating in DNA repair, apoptosis control and cell cycle progression [12–14]. *CHEK2* (22q12.1) encodes checkpoint protein kinase 2 (CHK2), which lies immediately downstream of the ATM protein kinase, encoded by the *ATM* gene (11q22.3). DNA damage is recognised by sensor proteins that recruit ATM, which is responsible for phosphorylating and activating CHK2, as well as other proteins involved in DNA repair [12, 13]. Once activated, CHK2 phosphorylates multiple target molecules, including TP53 (which can also be directly activated by ATM), KAP1, CDC25A, CDC25C and BRCA1 [12, 13].

Germline PVs in *CHEK2* usually affect the stability of the protein or its kinase activity [12]. The main associated risks include a moderate increase in the risk of breast cancer in women between 20% and 44% and an increased risk of colorectal cancer from 5% to 10% in life. Evidence also relates it to an increased risk of renal, thyroid and prostate cancer [6, 12, 14, 15]. On the other hand, Germline PVs in* ATM* are associated with a moderate increase in the risk of breast cancer in women of 20%–40% in life, pancreas of 5%–10%, ovarian of up to 3% and unspecified prostate cancer risk [6]. The functional deterioration of the same signaling pathway explains the phenotypic overlap in the neoplasms associated with each gene.

Theoretically, a potential synergistic effect could be postulated between both variants due to their participation in the same signaling pathway and the fact that CHK2 is an ATM effector. However, it could also be hypothesised that by participating in the same path and the direct functional relationship between both genes, the effect on the development of neoplasms will not necessarily be more severe since the compromised downstream effectors are largely similar.

In the previously reported and present case, the phenotype of most patients resembles the classical neoplasia spectrum of either of these moderate-penetrance breast cancer risk genes. In one case, there is likely only a manifestation of the *CHEK2* spectrum with colorectal cancer, and the appearance of bladder cancer as a second primary in one of the breast cancer patients could be considered an atypical manifestation. However, this assumption would need further confirmation of the involvement of the variants in the development of this tumour, along with an evaluation of environmental factors possibly related [8–10].

*CDKN2A* is a tumour suppressor gene associated with FAMMMS (OMIM #155601), melanoma-pancreatic cancer syndrome (OMIM #606719) and Melanoma and neural system tumour syndrome (OMIM# 155755). It encodes two proteins, p16 ^INK4A^, an inhibitor of cyclin-dependent kinase, and p14 ^ARF^, which binds to the p53-stabilising protein MDM2. p16 ^INK4A^ produces G1 cell cycle arrest by inhibiting retinoblastoma protein phosphorylation, and p14 ARF performs its p53-dependent function by binding and inhibiting MDM2 protein in both G1/S and G2/M phases [16–18]. *CDKN2A* PVs give a lifetime risk of 28%–76% for melanoma and an absolute risk of 15% for pancreas cancer [6].

Depending on the protein (either p14 or p16) affected, there are other cancer risks, such as head and neck squamous cell carcinoma, neural system tumours, gastrointestinal cancer, breast cancer and lung adenocarcinoma [19].

*BRCA1* is a tumour suppressor gene involved in the repair of DNA double-strand break repair through homologous recombination. PVs are associated with an absolute risk higher than 60% for female breast cancer, 39%–58% for ovarian cancer, 7%–26% for prostate cancer and up to 5% for pancreatic cancer [6].

These two genes participate in different pathways, indicating the possibility of an additive effect of independent risks for pancreatic cancer. As for female breast cancer, a recent meta-analysis carried out across three large whole-exome sequencing datasets pointed to an association between deleterious protein-truncating or missense variants in *CDKN2A* and breast cancer at exome-wide significance [20].

In this case, the phenotype resembles a typical presentation of *BRCA1-related* cancer with triple-negative breast cancer at a young age of onset. The family history in the maternal line also coincides with a *BRCA1* family history of cancer with high penetrance of breast cancer and male individuals with gastric cancer [21]. There was no history of pancreatic cancer, nervous system neoplasias or confirmed melanoma in either family nor did she present a typical skin phenotype of FAMMS. Nevertheless, *CDKN2A* PVs are sometimes found as a secondary finding in breast cancer panel testing, with its role in this type of cancer still poorly elucidated [20, 22], and some of the cancer cases in the paternal line of this patient could be related to a *CDKN2A* spectrum, with molecular confirmation needed.

Determining the phenotypic results of the association of variants in these gene combinations is essential as it may have consequences regarding prognosis, follow-up and treatment. However, due to the low number of reported cases, it is impossible to establish a personalised follow-up protocol based on available information.

In Case 1, even though the NCCN follow-up guidelines do not recommend performing a risk-reducing mastectomy in the contralateral breast, the proband decided to take that option to reduce her risk of cancer. Follow-up by colonoscopy was recommended, and pancreatic follow-up was not indicated due to the absence of a family history of this type of cancer [6, 7]. Regarding the gynecological follow-up related to the risk of ovarian cancer associated with *ATM*, it was indicated to continue gynecological check-ups for her abnormal uterine bleeding; however, in the context of a possible hysterectomy, the patient was considering performing a bilateral risk-reducing salpingo-oophorectomy. Despite the lack of evidence, the patient’s wishes to implement more drastic measures than those indicated theoretically for both genes suggest that this double genetic diagnosis can generate more fear or anxiety than usual cancer predisposition syndromes.

In the case of the patient with variants in *BRCA1* and *CDKN2A*, risk-reducing surgeries of the contralateral breast and bilateral salpingo-oophorectomy were scheduled following the *BRCA1* guidelines [6]; however, she had not yet been able to access dermatological and pancreatic screening in the public health system.

In both reported cases, it was not possible to carry out cascade family testing owing to economic reasons, which is a significant limitation in preventing risks in these families and collecting information related to the individual and combined phenotypes of these genes in these different people. Testing subjects at risk and cascade family testing access is still very limited in Chile, depending on the patients’ and families’ capacity for out-of-pocket paying [23, 24]. The prevalence of PVs in CSGs in the country and Latin America is still poorly characterised [25, 26], and local reports can help raise awareness of the presence of rare entities such as MINAS in our population and have a reference of its manifestations in the local genetic and environmental background.

## Conclusion

Carrying out functional studies and encouraging the reporting of other cases and their families is essential to establish specific risks for individuals with this syndrome as well as better follow-up guidance.

## Conflicts of interest

The authors have no relevant financial or non-financial interests to disclose.

## Funding

This study received no funding.

## Ethical approval

This study was performed in line with the principles of the Declaration of Helsinki. Approval was granted by the Healthcare Ethics Committee of FALP and carried out in compliance with the current legislation in Chile on Scientific Research in humans of laws 20,120 and 19,628. All participants provided informed consent for the publication of their clinical information. Participants provided informed consent for the genetic studies and the publication of their clinical data.

## Data availability

On request from the authors.

## Author contributions

All authors contributed to writing and reviewing the manuscript, which they then read and approved as final.
